# Trends on CO_2_ Capture with Microalgae: A Bibliometric Analysis

**DOI:** 10.3390/molecules27154669

**Published:** 2022-07-22

**Authors:** Alejandra M. Miranda, Fabian Hernandez-Tenorio, David Ocampo, Gabriel J. Vargas, Alex A. Sáez

**Affiliations:** 1 Biological Sciences and Bioprocesses Group, School of Applied Sciences and Engineering, Universidad de EAFIT, Medellín 050022, Colombia; ammirandap@eafit.edu.co; 2 Process Engineering Department, School of Applied Sciences and Engineering, Universidad EAFIT, Medellín 050022, Colombia; fehernandt@eafit.edu.co; 3 Industrial and Chemical Processes Group, School of Engineering, Universidad de Antioquia, Medellín 050010, Colombia; david.ocampoe@udea.edu.co; 4 I&D Cementos Argos S.A, Centro de Argos para la Innovación, Medellín 050022, Colombia; gvargasva@argos.com.co

**Keywords:** microalgae, CO_2_, bibliometric analysis, CO_2_ capture, biofuel

## Abstract

The alarming levels of carbon dioxide (CO_2_) are an environmental problem that affects the economic growth of the world. CO_2_ emissions represent penalties and restrictions due to the high carbon footprint. Therefore, sustainable strategies are required to reduce the negative impact that occurs. Among the potential systems for CO_2_ capture are microalgae. These are defined as photosynthetic microorganisms that use CO_2_ and sunlight to obtain oxygen (O_2_) and generate value-added products such as biofuels, among others. Despite the advantages that microalgae may present, there are still technical–economic challenges that limit industrial-scale commercialization and the use of biomass in the production of added-value compounds. Therefore, this study reviews the current state of research on CO_2_ capture with microalgae, for which bibliometric analysis was used to establish the trends of the subject in terms of scientometric parameters. Technological advances in the use of microalgal biomass were also identified. Additionally, it was possible to establish the different cooperation networks between countries, which showed interactions in the search to reduce CO_2_ concentrations through microalgae.

## 1. Introduction

The excessive release of greenhouse gases (GHG) and the increase in the concentration of carbon dioxide (CO_2_) (approximately 75% of greenhouse gases GHG) in the last two centuries [1] have aroused the attention of all countries, due to the serious threat they represent to the environment and human health [2,3]. Alarming data published by the International Energy Agency (IEA) in March 2021 report that, although there was a decrease of approximately 2000 million tons in 2020, emissions are increasing with greater intensity compared with the past several years, as the different economies struggle to recover after the crisis generated by COVID-19 [4].

According to the World Health Organization (WHO), it is estimated that seven million deaths a year are the product of environmental pollution arising from GHGs [5], and it is predicted that these deaths may amount to up to nine million in 2060 if the growing trend in CO_2_ and GHG emissions continues [6]. The United Nations Development Program (UNDP) reveals that 91% of geophysical disasters are caused by climate change, and according to The Economist Intelligence Unit of the United States, it is estimated that, if adequate measures are not taken to combat this problem, climate effects can cost the world 7.9 trillion dollars and cause the world economy to lose 3% of gross domestic product (GDP) by 2050 [7].

It is known that drastic climate change affects the economic growth of the world, manifesting in adverse effects in different sectors such as agriculture, with a reduction in agricultural productivity, sanctions and restrictions on industries due to the high carbon footprint, and interruptions in tourism activities [8,9]. Hence, it is necessary that, through bureaucratic processes, policymakers join efforts to help reduce the environmental problem of CO_2_ emissions [10].

Among the different technologies for capturing CO_2_, there are microalgae, which are photosynthetic microorganisms that can naturally fix CO_2_ from 10 to 50 times that of terrestrial plants to produce O_2_. In addition, these microorganisms are capable of generating added-value products of interest to the pharmaceutical, biomedical, cosmetic, chemical and nutraceutical industries [11]. For example, compounds with antioxidant, anticancer, and antimicrobial activities, including carotenoids, polysaccharides, lipids, phenolic compounds, vitamins, and peptides, have been extracted from different species of microalgae [12,13,14]. They are also used to produce biofuels, such as biocrude oil, biodiesel, biogas, and hydrogen [15,16,17,18]. Likewise, studies on the production of bioplastics using microalgae as an alternative to the use of petroleum-derived polymers have been reported [19,20,21]. Therefore, CO_2_ capture systems with microalgae can be economic levers that enable the commercialization of these products while contributing to the mitigation of high concentrations of CO_2_ on the planet [22,23,24].

The authors of [25] suggested that biofuels obtained from microalgae could be commercially available by 2020–2025 due to increased research. However, authors of other studies, such as [26,27], stated that research should be intensified in the area of carbon capture, carbon sequestration, the use of SCO products from the microalgae chain, and the use of wastewater as a growth medium to achieve competitive energy products in the market. The current state of microalgae research includes projects such as the industrial-scale demonstration of sustainable algae cultures for biofuel production [28], which sought to produce biofuels on a large scale using low-cost microalgae species. This project reported values of 200 tons/acre of biomass with a net oil content of up to 20%. Another project was improving photosynthetic solar energy conversion in microalgal cultures for the production of biofuels and high-value products, also called SOLENAL-GAE, funded by the European Research Council (ERC) [29]. This project investigated how to improve biomass production in microalgae by making the process of converting light energy to biomass more efficient. The project achieved the design of microalgae strains with improved photosynthetic efficiency, allowing yields of up to 30% in biomass production. International commercialization of innovative products based on microalgae was a project that validated the real environment and the development of AlgaEnergy’s production facilities from TRL7 to TRL9, i.e., converting a demonstration plant to a commercial industrial scale [30].

Although microalgae constitute a strategy with a high potential for CO_2_ capture, there are still challenges regarding the use of microalgal biomass, the evaluation of the life cycle of microalgae technologies, and the execution of policies on CO_2_ mitigation. Therefore, this article aims to present a comprehensive review on obtaining energy products from microalgal biomass, analyze the life cycle of CO_2_ capture technologies, and identify current policies for CO_2_ reduction with microalgae. In addition, this review focuses on providing a bibliometric analysis on the capture of CO_2_ with microalgae, in which trends are indicated on energy products, strains used with greater interest, scientific production, and the use of biomass in applications of interest. The analysis presented here represents an important contribution from the bibliometric approach that could be positive for the development of research on CO_2_ capture with microalgae and could provide some suggestions to researchers working on the subject.

## 2. Bibliometric Analysis for CO_2_ Capture with Microalgae

The problems caused by contamination by CO_2_ generate interest worldwide because they are constantly worsening; for this reason, it is pertinent to analyze the trend regarding the capture of CO_2_ with microalgae through a systematic search in the Scopus scientific database with the equation TITLE-ABS KEY (“microalgae” AND “capture” AND (“CO_2_” OR “carbon dioxide”)) AND (LIMIT-TO (DOCTYPE, “ar”) OR LIMIT TO (DOCTYPE, “re”)), which compiled information from 383 documents taking into account research and review articles. Bibliometric parameters such as the total number of citations, average number of citations per article, categorization of publications with the highest citation, and the process of evolution of the research topic were determined from the Bibliometrix package of the R commander software (x64 4.1.0). Additionally, bibliometric maps were developed through co-occurrence and co-authorship analyses using the VOSviewer version 1.6.16 software. It is noteworthy that the compiled documents were filtered in order to avoid the repetition of terms with abbreviations and hyphens [31].

**Figure 1:**
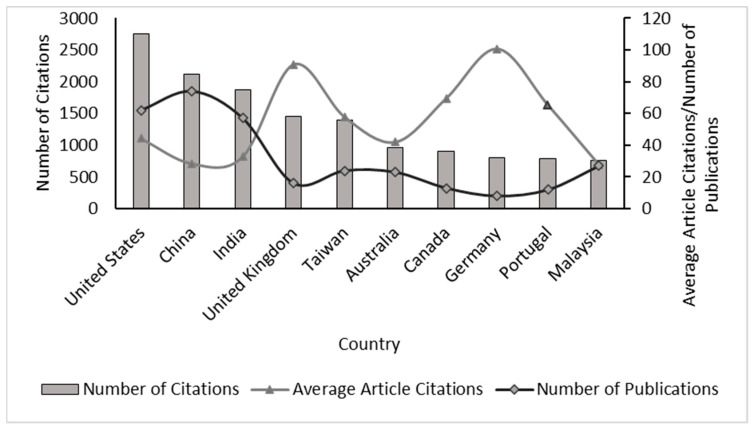
Leading countries in publications on CO_2_ capture with microalgae, data-based Scopus reports.

The bibliometric analysis allowed us to analyze the relevance of the research published between 1993 and 2022 on the capture of CO_2_ with microalgae; consequently, the results showed the categorization of the leading countries in article publications on the subject (Figure 1), finding that the United States presents the highest number of citations (2749), followed by China (2117). In addition, it was identified that Germany showed the highest value in average citations per article (100.50) and the lowest number of documents (8), compared with the other countries; therefore, the impact of these publications in the area of study and their possible use as important references for other research studies is evident. It is noteworthy that the information extracted from Scopus was categorized according to the author’s affiliation address correspondent.

Table 1 presents the most cited publications related to CO_2_ capture with microalgae, with the listed documents corresponding to review articles that are key reference works on the subject. In addition, the contribution with the greatest impact was found to have 751 citations and 93.87 citations per year, made by authors from research centers in the United Kingdom [32]. In this review study, the environmental impacts of carbon capture and storage technologies were discussed, suggesting that the synergy between carbon capture, storage, and utilization strategies may enable climate change mitigation, in conjunction with other perspectives such as renewable energies, energy demand reduction, and the use of other low-carbon technologies. However, the results also revealed the document with the highest number of citations per year (98.00) that is used for its significant contributions to the capture of CO_2_ with microalgae [33], from the analysis of which several technological perspectives and economies stand out for the use and elimination of CO_2_; in this study, 10 strategic routes are proposed for the use of CO_2_—namely, the development of chemical products based on CO_2_, microalgae fuels, concrete construction materials, fuels based on CO_2_, bioenergy with CO_2_ capture and storage, enhanced oil recovery with CO_2_, forestry/reforestation techniques, enhanced weathering, biochar, and land management through soil carbon sequestration techniques.

**Figure 2:**
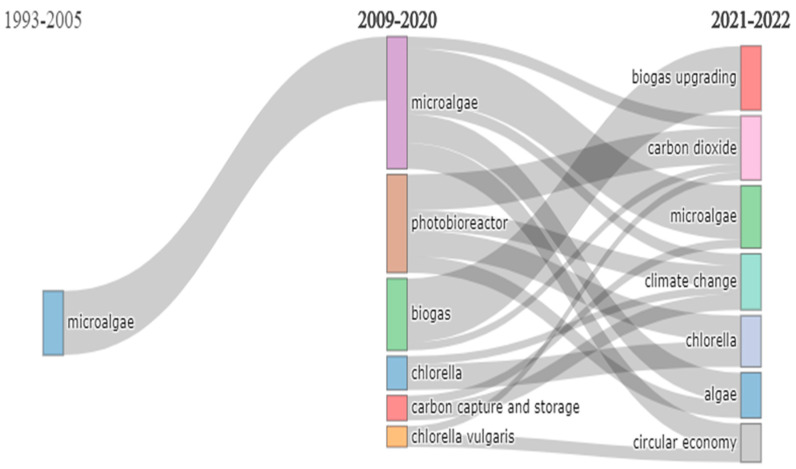
Map of thematic evolution of keywords on CO_2_ capture with microalgae (Bibliometrix).

Furthermore, a thematic evolution map was obtained through Bibliometrix, which describes the development of keywords over time in research on CO_2_ capture with microalgae (Figure 2). The map was made considering three different stages (1993–2005, 2009–2020, and 2021–2022) and showed that, between 1993 and 2005, research on the subject was limited with six publications made. As for the other stages, the progress of the topic of CO_2_ capture with microalgae was identified, for example, between 2009 and 2020, for which keywords related to the production and use of microalgal biomass were found. Most recently, from 2021 to 2022, the integration of the term circular economy was observed, with the purpose of establishing microalgae as sustainable systems that promote energy efficiency while preventing environmental deterioration.

**Figure 3:**
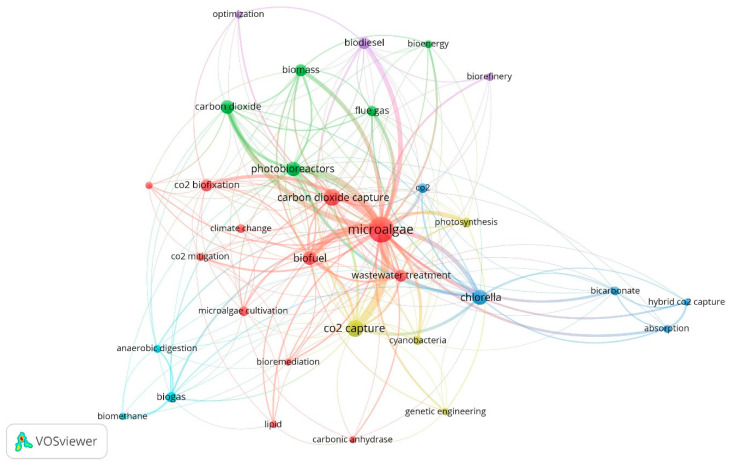
Bibliometric map of author keywords in publications on CO_2_ capture with microalgae. Six theme groups: yellow, blue, green, purple, red, and sky blue.

Figure 3 shows the bibliometric map of author keywords obtained through co-occurrence analysis, according to which six groups of interrelated keywords were identified. The grouped themes are associated with energy products such as biogas, biodiesel, lipids, biofuel, and biorefinery, which show the approach toward the use of microalgal biomass as an alternative to meet energy demands worldwide (light blue, red, and purple node). Similarly, terms associated with wastewater treatment and bioremediation were found (red node); in addition, the *Chlorella* genus was identified on the map, possibly because it is an attractive genus for obtaining biofuels due to its lipid content (39.4–41.2%) [42]. From a broader perspective, it was possible to establish the main keywords reported based on their co-occurrence: microalgae (183), CO_2_ capture (53), carbon dioxide capture (52), *Chlorella* (41), and photobioreactors (33) were the words used with greater proximity in the published documents according to the data from Scopus reports.

**Figure 4:**
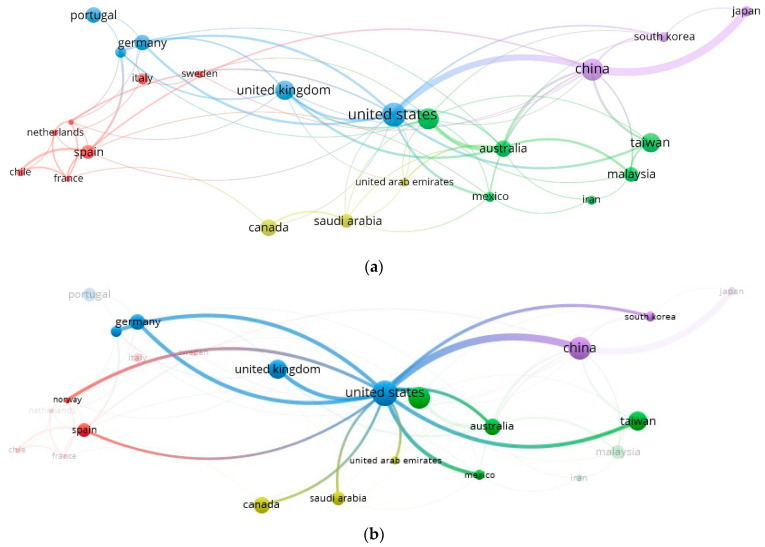

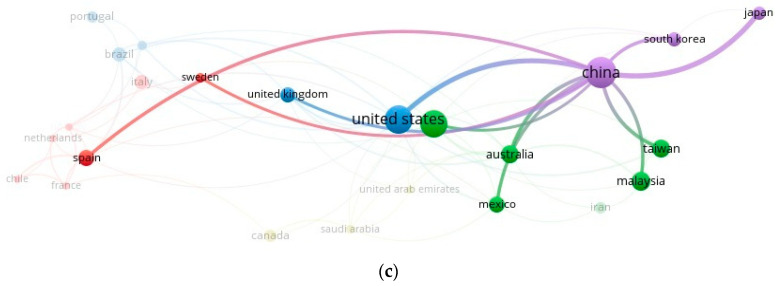
Collaboration maps of countries in publications on CO_2_ capture with microalgae: (**a**) general collaboration network; (**b**) United States collaboration network; (**c**) China collaboration network. Five groups of countries: red, green, purple, yellow, and blue.

Additionally, the analysis made it possible to build collaboration networks of countries on CO_2_ capture studies with microalgae (Figure 4a), finding five interconnected groups (red, blue, green, yellow, and purple), with the United States as the country with greater cooperation in the investigations carried out, which is in accordance with the registered result of a greater number of citations (2749). The strongest network made up of 15 countries also revealed the United States as the main node (Figure 4b). China was identified as constituting the second solid network of collaboration, with 12 countries (Figure 4c). It is noteworthy that China was the country with the highest number of published documents (74), so it was expected that it would be the main node of the cooperative network. The presented results showed the relationship that exists between researchers and their respective institutional affiliations; therefore, the scientific cooperation in the search to reduce CO_2_ concentrations with microalgae systems and leverage with economic sectors for the use of biomass were highlighted.

## 3. Value-Added Energy Products

The depletion of fossil fuels and population growth are exacerbating the energy crisis and accelerating interest in the search for alternative energies to meet demands and avoid possible global chaos [43]. Within this search, obtaining energy from microalgal biomass emerges not simply as an alternative to contribute to reducing the energy crisis but to help reduce the negative impacts generated by excessive GHG pollution in the environment [44]. In our bibliometric analysis, a tendency was found toward research related to obtaining products with an energetic nature such as biodiesel, biogas, biocrude, and biofuels from CO_2_ capture processes using microalgal culture systems.

The composition of the biomass and the conditions of the technology to be used play important roles in increasing the production of the energy product of interest. For example, for the production of biodiesel, biomass with a high lipid content must be obtained, and this can be achieved by modifying factors such as temperature, lighting, pH, CO_2_ supply, and the number of nutrients in the culture medium [45,46,47]. Anh [48] reported that a concentration of 401.5 ± 47.3 mg/L of fatty acids can be obtained for *Nephroselmis* sp., and the biodiesel obtained from this strain can reach cetane index values of 52.31, indicating that this biomass can be considered for the production potential of biodiesel. However, even if a biomass product with a high lipid content is obtained, the harvesting and drying of the biomass increase production costs and make biodiesel a non-competitive product relative to other raw materials [49,50], so it is required to reduce costs in cropping systems and harvesting biomass, to improve the economic viability of these systems [51,52].

On the other hand, the biogas obtained from microalgae biomass is considered another high-value energy product [53]. Through a life cycle evaluation, Sun [54] reported that the biogas obtained from microalgal biomass through anaerobic digestion at room temperature and without additional dehydration can present more energy, compared with biodiesel; however, microalgae cells inherently have compact cell walls, which leads to low biogas yield. Xiao [55] reported that all biomass carbohydrates, lipids, and proteins can be converted into biogas and achieve theoretical yields of up to 0.85 L/g of volatile solids; however, the experimental yield only reached values of 0.28 L/g. g of volatile solids.

Other microalgae conversion technologies include hydrothermal liquefaction (HTL) [56,57], which is considered a promising technology for microalgae conversion [58,59] because it does not require catalysts or organic solvents [60] and can convert all biomass (proteins, lipids, and carbohydrates) into different energy products from finely adjusted operating conditions, which expands the applications of biomass [61,62] and the direct use of wet biomass [63]. Due to these advantages, the costs of production in terms of energy consumption of biomass treatment were reported by Ren [64] using simulation results, which estimated that, by applying HTL, about 3 MJ of energy can be saved compared with the use of solvent extraction technologies (energy consumed during drying); in addition, sweet water consumption was reduced by 33%, and the demand for nitrogen and phosphorus by 44% [65]. However, one of the main disadvantages of this technology is the presence of heteroatoms such as nitrogen (7%), oxygen (9%), and sulfur (1.5%) in the biocrude, reducing quality, generating thermal instability, sediments, and rubber [66,67]. Therefore, to solve these problems, authors such as Tang [68] and Huang [69] proposed strategies such as the evaluation of different catalysts or the two-step approach for HTL. Miranda [70] reported that, by varying the nitrogen levels in the culture medium, a biocrude with nitrogen contents between 2.8% and 6.7% and yields between 18.9% and 49.1% can be obtained.

Although biofuels obtained from microalgal biomass have many advantages, it is important to bear in mind that their production faces difficulties in biomass harvesting, which increases the energy cost and production of the energy product; therefore, it must be followed in the search for alternatives that allow reductions in costs, such as the use of wastewater, reuse of nutrients from different processes, etc., in order to increase the profitability of the production of energy products [71,72,73].

## 4. Life Cycle in CO_2_ Capture with Microalgae

A valuable tool to determine the environmental impacts associated with a biofuel product system and production pathways in order to increase its market value is the life cycle assessment [74,75]. Currently, high-value-added products are obtained from microalgal biomass, and through these systems, it is possible to capture GHG, especially CO_2_ [76]; however, the production of biofuels from microalgae is still an uncompetitive technology in the market relative to fossil fuels, due to the high energy consumption of harvesting, oil extraction, and biomass transformation [77,78].

The evaluation of the different options of crops and harvests for the production of microalgal biomass allows for achieving a reduction in the critical stages in the production of biomass. For example, Pankratz [79] evaluated the environmental performance of HTL and the pyrolysis of microalgae to produce diluents, reporting that the HTL process shows an environmental benefit (10.20–45.65 g CO_2_-eq.MJ^−1^) possibly due to the processing of wet biomass, which does not result in energy requirements and GHG emissions due to the drying of biomass. Derose [80] studied the economic viability of the conversion of microalgae through two chemical routes to produce biofuels, and the results indicated a minimum fuel sale price of 3.39 dollars/L and 2.75 dollars/L for the biochemical and thermochemical routes, respectively. In addition, their study showed that the minimum fuel sales price can be reduced by lowering the cost of biomass feedstock, and ash content, and improving HTL fuel yields [81].

Despite the potential of energy products obtained from microalgal biomass and the ability of life cycle analysis to help understand the potential environmental impact of these processes, analyses that include scaling and promising techniques such as HTL are scarce in the literature [82]. Therefore, it is recommended to continue and strengthen research on these processes to minimize the gaps between production costs, profitability, and environmental benefits.

## 5. Research Opportunities in CO_2_ Capture with Microalgae

Biological technologies are considered tools that contribute to the mitigation of CO_2_ emissions, among which microalgae crops emerge as promising alternatives that not only allow the capture of GHG but also provide a wide range of value-added products and biofuels [83]. However, although several companies, institutes, and governments invest in these technologies, the commercial production of biofuels is limited [84,85,86]. One reason is the economic feasibility of large-scale biomass production, which remains a major obstacle to its commercialization [87]. Consequently, different research approaches have been generated to reduce the gaps and propose processes that improve the competitiveness of these technologies. Some of the approaches include mixotrophic cultures, which provide a source of organic carbon in addition to inorganic carbon that would favor increased cell growth and productivity [88]. Another strategy is the reuse of wastewater from industrial processes because they are considered promising sources for the supply of nutrients, thus leading to a reduction in the costs of the culture medium (phosphorus, nitrogen, metals) [89]. Moreover, these processes provide the opportunity to reduce pollution due to water generated by the industries. For instance, Kholssi [90] reported the removal of 50–95% of nitrogen and 50–80% of phosphorus in wastewater by using *Chlorella vulgaris* cultures.

Additionally, the combination of microalgae and bacteria is presented as an efficient, economical, and sustainable methodology for the production of biohydrogen, compared with monoculture, because the mutual exchange of nutrients and electrons facilitates the production of hydrogen. However, for production on an industrial scale, the operating conditions must be standardized, which constitutes a research opportunity to achieve an improved and sustainable production in the long term through the use of a wide range of sources of wastewater [91].

The cultivation of cyanobacteria and microalgae in support of human habitation on Mars is one of the most challenging hypotheses proposed. The remoteness of the planet and the inhospitable conditions of the surface (low pressure, low temperatures, and absence of basic resources such as water) pose challenges for the growth of these microorganisms [92]. For instance, Frackrell [93] reported that microalgae can serve as regolith soil biofertilizers for the production of food for astronauts. Fields [94] indicated that, in addition to synthesizing the native forms of antiviral, anticancer, and antibacterial agents as secondary metabolites, microalgae can serve as agents to produce other compounds with medical importance, in addition to transforming CO_2_. Due to the above, it is possible to suggest that research is still required regarding the improvements of microalgae cultures and CO_2_ capture, which allow us to optimize processes and contribute to the reduction in environmental pollution that is constantly worsening.

## 6. Global Microalgae Market

The microalgae industry produces approximately 7000 tons of dry biomass each year, reflecting a global market, with revenues of USD 3.8 to 5.4 billion [95]. These figures indicate the interest of the different industrial sectors in using biomass as a safe and sustainable strategy. Currently, there are known government programs that have allowed the development of research to combat global warming [96]. In addition, there are companies or organizations in charge of developing products and technologies based on microalgae, among which the company ExxonMobil stands out, with projections of 10,000 barrels per day of microalgae biofuel for 2025 [97]. También hay empresas como Ecoduna, Cellana, Algomed, Algabt, Earthrise Califor-nian Spirulina, entre otras, que producen productos de microalgas con aplicaciones nutracéuticas y medicinales. Other companies such as Carbon BioCaptur, Helios NRG, PureBiomass, and Algoliner are interested in developing technologies for the improvement of microalgae cultivation systems (Table 2).

The production and commercialization of microalgae are led by Asian countries such as China and Japan. For example, China, in terms of microalgal biomass storage and harvesting, has been one of the main producers in the last 15 years. Biomass utilization in China is focused on obtaining high-value by-products with applications in the food, pharmaceutical, and cosmetics industries. In comparison, in Japan, the production of microalgae focuses on biofuels, with an approximate market of USD 1.17 billion [98]. On the other hand, the European algal sector is distributed in 43% of companies producing microalgae, and 57% is focused on marine algae. In addition, Europe is estimated to be the leader in the Spirulina market, and it is known that the largest volumes of microalgal biomass are produced in countries such as Ireland, Norway, and France [99].

**Table 2:** Microalgal commercial producers.

Company	Applications	Developments and Projections	References
ExxonMobil	Biofuels	10,000 barrels of microalgae biofuels per day by 2025	[97]
Ecoduna	Nutraceutical	Increase production mainly in the food sector	[100]
Heliae	Agriculture	PhycoTerra^®^ products	[101]
*Chlorella* Industry Co., LTD	Medical, pharmaceutical, and nutritional science	Manufacture and sales of *Chlorella* products	[102]
Cellana	Human health, biofuel feedstock, and animal health	ReNew™ products	[103]
PureBiomass In	Cultivation technologies	Development of cultivation systems for microalgae	[104]
Algoliner	Cultivation technologies	Development of cultivation systems for microalgae	[105]
Algomed	Nutraceutical	Manufacture and sales of *Chlorella* products	[106]
Helios NRG	Technology development and consulting group	Development of cultivation systems for microalgae	[107]
Carbon BioCaptur	Technology development and consulting group	Development of cultivation systems for microalgae	[108]
Algabt	Carotenoid production	Development of bioproducts for nutraceutical, cosmetic, pharmaceutical, and aquaculture markets.	[109]
Earthrise Californian *Spirulina*	Nutraceutical	Manufacture and sales of *Spirulina* products	[110]

## 7. Conclusions and Future Outlook

Data mining is characterized as a tool that offers the possibility of extracting patterns from large databases, with the purpose of providing evidence of the impact of research results. Consequently, this bibliometric analysis revealed trends in technological advances related to the use of microalgal biomass to obtain energy products such as biofuels. In addition, scientometrics showed the evolution of CO_2_ capture research with microalgae, finding significant growth in the United States, China, India, and European countries. It is noteworthy that the countries with the largest populations and the highest CO_2_ emissions to the atmosphere were those that showed the greatest scientific production on the subject. This provides an encouraging outlook in the search for alternatives to conventional processes in which microalgae can favor the reduction in global warming and the development of products with a zero-carbon footprint. It was also evident that microalgae studies in the 1990s were not funded on the same scale as they are today. This is because increased pollution has made it possible to increase microalgae research, which is encouraged by government programs due to the concerns of nations about climate change and the scarcity of conventional energy sources. Additionally, in this study, the incorporation of new approaches such as the circular economy was highlighted, which contributes to the management of sustainable and competitive processes that combine the objective of reducing carbon emissions and the use of waste.

Although microalgae have been established as an ecological raw material, the challenges for the commercialization of microalgae systems for capturing CO_2_ and generating value-added products show the need to redesign production patterns and optimize and integrate processes in order to overcome limitations on economic viability. In addition, more research efforts and investments in different fields of knowledge are required, from the biological, biochemical, and engineering perspectives, among others, which, together with adequate policies, will attract sectors of the industry that favor the expansion of the global market for microalgae technologies.

## Figures and Tables

**Table 1:** Documents with the highest citations in global research on CO_2_ capture with microalgae.

Title	Journals	Authors Affiliation Countries	Number of Citations	Number of Citations per Year	References
Carbon capture, storage, and utilization technologies: A critical analysis and comparison of their life cycle environmental impacts	*Journal of CO_2_ Utilization*	United Kingdom	751	93.87	[32]
The technological and economic prospects for CO_2_ utilization and removal	*Nature*	Germany, United Kingdom, States United	393	98.00	[33]
Prospects of biodiesel production from microalgae in India	*Renewable and Sustainable Energy Reviews*	India	388	27.71	[34]
Perspectives on microalgal CO_2_-emission mitigation systems—A review	*Biotechnology Advances*	Taiwan	382	31.83	[35]
Cyanobacteria and microalgae: A positive prospect for biofuels	*Bioresource Technology*	India, Suiza	379	31.58	[36]
Integrated CO_2_ capture, wastewater treatment, and biofuel production by microalgae culturing—A review	*Renewable and Sustainable Energy Reviews*	Saudi Arabia, Canada	349	34.90	[37]
Carbon dioxide capture from flue gases using microalgae: Engineering aspects and biorefinery concept	*Renewable and Sustainable Energy Reviews*	Portugal	283	25.72	[38]
Resource demand implications for US algae biofuels production scale-up	*Applied Energy*	United States	247	20.58	[39]
Closed photobioreactors for the production of microalgal biomasses	*Biotechnology Advances*	Canada	245	22.27	[40]
Microalgae: The potential for carbon capture	*BioScience*	United States	243	18.69	[41]

Data based on Scopus reports.

## Data Availability

Not applicable.

## Sample Availability

Samples of the compounds are not available from the authors.
